# First isolation of *Rickettsia amblyommatis* from *Amblyomma mixtum* in Colombia

**DOI:** 10.1186/s13071-023-05950-7

**Published:** 2023-09-20

**Authors:** Jenny J. Chaparro-Gutiérrez, Leidy Y. Acevedo-Gutiérrez, Nicole L. Mendell, Laura N. Robayo-Sánchez, Arlex Rodríguez-Durán, Jesús A. Cortés-Vecino, Diana Fernández, Alejandro Ramírez-Hernández, Donald H. Bouyer

**Affiliations:** 1 Research Group CIBAV, University of Antioquia, UdeA, Medellín, Colombia; 2 Department of Agricultural Sciences, Faculty of Veterinary Medicine, Lasallian University Corporation (Unilasallista), GIVET Research Group, Caldas, Antioquia Colombia; 3 Department of Pathology, University of Texas Medical Branch, 301 University Boulevard, Galveston, TX 77550 USA; 4 Research Group Veterinary Parasitology, Laboratorio de Parasitología Veterinaria, Universidad Nacional de Colombia, UNAL, Bogotá, Colombia; 5 Universidad de La Salle, Bogotá, D.C. Colombia

**Keywords:** Arthropods, Ticks, Colombia, Disease, *Rickettsia*

## Abstract

**Background:**

Rickettsiae are obligate intracellular Gram-negative bacteria that are the causative agent of rickettsioses and are spread to vertebrate hosts by arthropods. There are no previous reports of isolation of *Rickettsia amblyommatis* for Colombia.

**Methods:**

A convenience sampling was executed in three departments in Colombia for direct collection of adult ticks on domestic animals or over vegetation. Ticks were screened for the presence of *Rickettsia* spp. by real-time polymerase chain reaction (qPCR) amplifying the citrate synthase gene (*gltA*), and the positive sample was processed for isolation and further molecular characterization by conventional PCR. The absolute and relative frequencies were calculated for several tick species variables. All products from conventional PCR were further purified and sequenced by the Sanger technique. Representative sequences of 18 *Rickettsia* species were downloaded from GenBank. Consensus phylogenetic trees were constructed for the *gltA*, *ompB*, *ompA*, and *htrA* genes with 1000 replicates, calculating bootstrap values through the maximum likelihood method and the generalized time reversible substitution model in the MEGA 7.0 software program.

**Results:**

One female *Amblyomma mixtum* collected on vegetation was amplified by qPCR (*gltA*), indicating a frequency of 1.6% (1/61) for *Rickettsia* spp. infection. Sequence analysis of a rickettsial isolate from this tick in BLASTn showed 100% identity with *gltA* (340 base pairs [bp]), 99.87% for *ompB* (782 bp), 98.99% for *htrA* (497 bp), and 100% for *ompA* (488 bp) to *R. amblyommatis*. Concatenated phylogenetic analysis confirmed these findings indicating that the isolate is grouped with other sequences of *Amblyomma cajennense* complex from Panama and Brazil within the *R. amblyommatis* clade.

**Conclusions:**

This paper describes the isolation and early molecular identification of a *R. amblyommatis* strain from *A. mixtum* in Colombia.

**Graphical Abstract:**

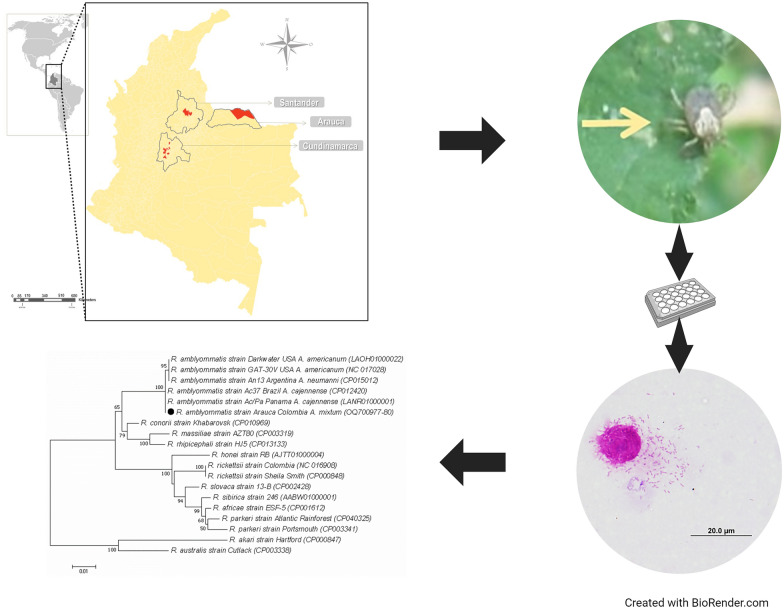

**Supplementary Information:**

The online version contains supplementary material available at 10.1186/s13071-023-05950-7.

## Background

Rickettsiae are obligate intracellular Gram-negative bacteria that cause rickettsioses and are spread to vertebrate hosts by arthropods such as ticks, fleas, lice, and mites [1]. A basal ancestral group, the typhus group, the transitional group, the spotted fever group (SFG), and the Tamurae/Ixodes Group (TIG) [2] are the five antigenic and genetic subgroups of rickettsiae [3, 4]. Today, the classification of new *Rickettsia* spp. strains is done by comparing their genomic sequences to the genome data of known strains [5]. Modern molecular techniques developed over the past few decades have made it easier to characterize new rickettsial species and have given rickettsiologists a chance to review some of the members of the genus [3, 4].

The field of rickettsiology made significant strides during the first two decades of the twenty-first century, and today, more than 15 *Rickettsia* species, along with several other unidentified members of this bacterial genus, are known to exist in the Neotropical region, primarily linked to ticks [6, 7]. *Rickettsia bellii* and *Rickettsia amblyommatis* have been found in numerous tick species [8–10]. These rickettsial species have been discovered in almost every tick species in the Neotropics, revealing a wide phylogenetic diversity of ticks and molecular differentiation of *R. amblyommatis* strains [11]. Although *R. amblyommatis* is widely distributed in the Neotropical region, it has not yet been determined whether it is a pathogen for vertebrates.

However, human clinical and serological evidence indicates that *R. amblyommatis* elicits a mild immune response [12, 13], with a macular rash at the tick bite site [14]. In animals, studies in guinea pig and mice models for *R. amblyommatis* infection elicited different immune responses. The guinea pig models presented several clinical outcomes ranging from mild symptoms of the disease [15, 16] to severe vascular inflammation [17], whereas the immune response in mice was acute, with a significant loss of body weight during the first days after *R. amblyommatis* infection [18]. On the other hand, there is evidence that infection with *R. amblyommatis* can induce cross-protection against transmission of pathogenic rickettsiae such as *Rickettsia rickettsii* or *Rickettsia parkeri*; hence, the presence of *R. amblyommatis* in the region may modify the epidemiology and severity of SFG rickettsioses [15, 17].

This paper describes the isolation and early molecular identification of a *R. amblyommatis* strain from *Amblyomma mixtum* in Colombia. This discovery will help to promote research comparing *R. amblyommatis* genome sequences in South America, which will provide insights into the molecular and genetic processes of reductive genome evolution in *Rickettsia*, their interactions with other bacterial pathogens of medical importance, their adaptation to new tick vectors and environments, and their impact on vectorial competence and disease transmission.

## Methods

### Sample collection

**Fig. 1:**
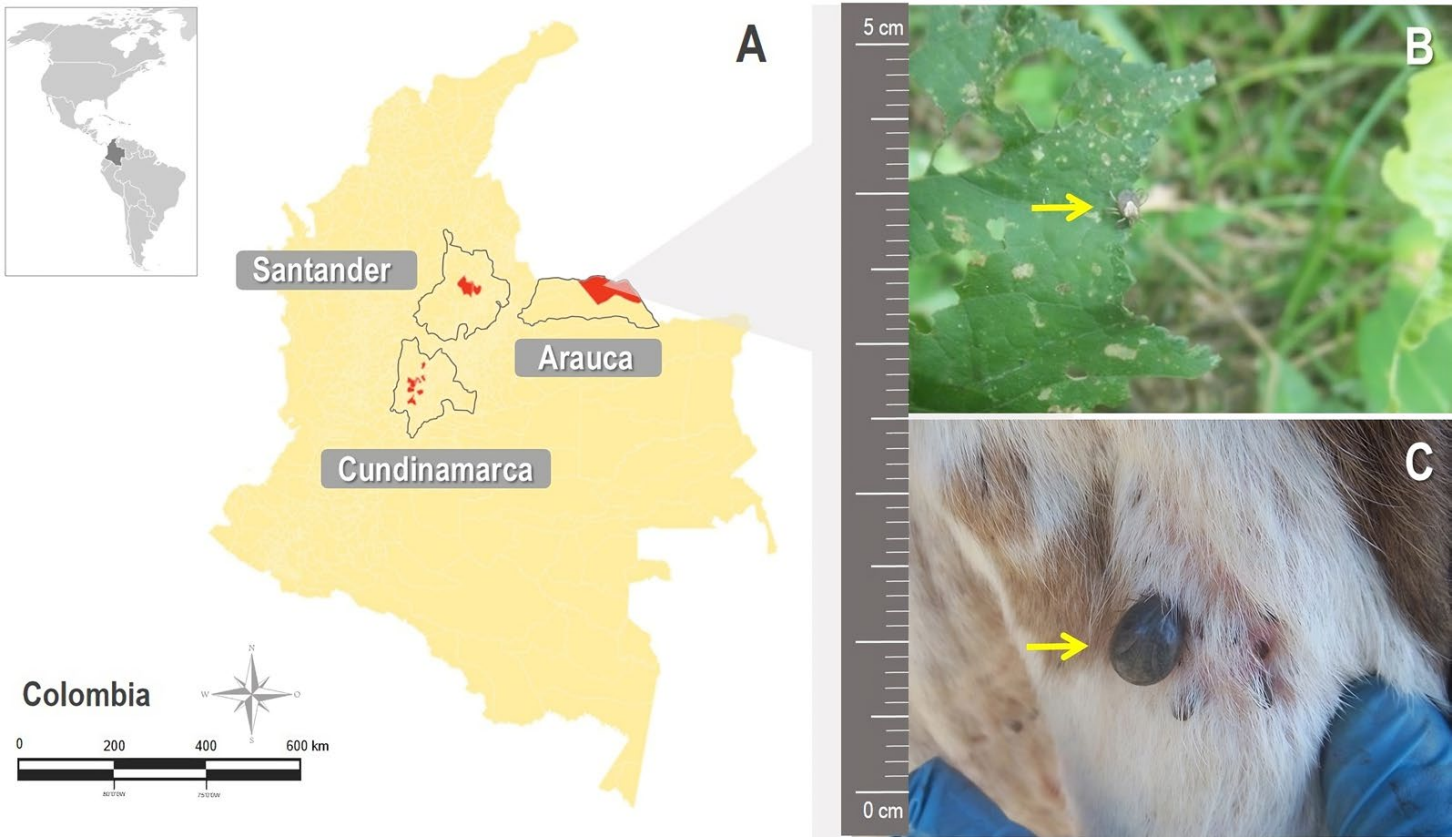
**A** Municipalities sampled (in red) in three departments of Colombia (QGIS v3.22.5.). **B** Adult of *Amblyomma mixtum* in non-parasitic phase collected on vegetation in the department of Arauca (Colombia) and **C** Adult of *A. mixtum* in parasitic phase collected from cattle in the department of Arauca (Colombia)

A descriptive study was performed during 2019 and 2020 in the departments of Arauca, Santander, and Cundinamarca in Colombia. The collection was approved by the Colombia National Environmental Licensing Authority (ANLA) no. 0255, March 14, 2014, and the Bioethics Committee FMVZ-UNAL (Facultad de Medicina Veterinaria y de Zootecnia–Universidad Nacional de Colombia) CB-088–2015. The sampling locations are shown in Fig. 1, and their specific data are summarized in a supplementary table (Additional file 1: Table S1). Convenience sampling was executed for direct collection of adult ticks in domestic animals (dogs, horses, cattle, or cats) or over vegetation, through flagging and dragging, with inspection every 10 m. The taxonomic status was defined according to Barros-Battesti for adults [19, 20], and one leg was removed for rickettsial screening. The classified samples were stored at −80 °C until further processing.

### Molecular and phylogenetic analysis

DNA was extracted from tick legs using Quick-DNA MiniPrep Plus Kit (Zymo Research, Irvine, CA, USA) according to manufacturer indications. The presence of inhibitors in the DNA was evaluated by amplification of the tick 16S ribosomal RNA (rRNA) constitutive gene (Table 1) [21]. The detection of *Rickettsia* spp. DNA was done by quantitative real-time polymerase chain reaction (qPCR) for screening of the citrate synthase gene (*gltA*) using CS5 and CS6 primers (Table 1). Also, conventional PCR was performed for the characterization of several genes using the primers presented in Table 1.

**Table 1:** List of primers used for real-time and conventional PCR

Gene	Primer name	Sequences	Size (bp)	Annealing (°C)	References
16S mitochondrial rRNA (16S mt rRNA)	16+	CCGGTCTGAACTCAGATCAAGT	472	50	Black and Piesman [22]
	16−	GCTCAATGATTTTTTAAATTGCTGTGG			
Citrate synthase (*gltA*)	CS5	GAGAGAAAATTATATCCAAATGTTGAT	147	60	Guedes et al. [23]; Labruna et al. [24]
	CS6	AGGGTCTTCGTGCATTTCTT			
	Probe	6-FAMd (CATTGTGCCATCCAGCCTACGGT) BHQ-1 3			
	CS78	GCAAGTATCGGTGAGGATGTAAT	401	48	Labruna et al. [24]
	CS323	GCTTCCTTAAAATTCAATAAATCAGGAT			
Outer-membrane protein B (*ompB*)	120-M59	CCGCAGGGTTGGTAACTGC	862	54	Roux and Raoult [25]
	120-807	CCTTTTAGATTACCGCCTAA			
Outer-membrane protein A (*ompA*)	190-70p	ATGGCGAATATTTCTCCAAAA	631	46	Regnery et al. [26]; Roux et al. [27]
	190-701	GTTCCGTTAATGGCAGCATCT			
17 kDa surface antigen (*htrA*)	17KdsF	GCTCTTGCAACTTCTATGTT	439	56	Webb et al. [28]
	17KdsR	CATTGTTCGTCAGGTTGGCG			

*Rickettsia sibirica* DNA and molecular-grade water were used as positive and negative controls, respectively. The positive tick sample identified by screening was processed for isolation.

The absolute and relative frequencies were calculated for several tick species variables, including place of collection, sex, source, and positivity. Positivity was defined as a sample with amplification of the *gltA* gene by PCR. All products from conventional PCR were further purified with ExoSAP-IT (Applied Biosystems, Waltham, MA, USA) and sequenced in triplicate by the Sanger technique using an ABI Prism 3130xl Genetic Analyzer (Applied Biosystems, Waltham, MA, USA) at the University of Texas Medical Branch (UTMB) Sequencing Core facility. The resultant electropherograms were manually edited, compared, aligned, and analyzed using the Nucleotide Basic Local Alignment Search Tool (BLASTn; https://blast.ncbi.nlm.nih.gov/Blast.cgi) and the ClustalW algorithm in MEGA 7.0 software [29, 30]. Representative sequences for *gltA*, *ompB*, *ompA*, and *htrA* genes of nine *Rickettsia* strains with complete genomes were downloaded from GenBank. Gene sequences from the same strain were used for each gene. Individual sequences were aligned with ClustalW, and consensus sequences were subsequently manually formed for each species. The best substitution model was selected in MEGA 7.0. Consensus phylogenetic trees were constructed for *gltA*, *ompB*, *ompA*, and *htrA* genes with 1000 replicates, calculating bootstrap values through the maximum likelihood method in MEGA 7.0 software.

### *Rickettsia* isolation

Frozen *A. mixtum* tick samples were allowed to warm at room temperature and placed in a 0.2 µm bottle top filter (Corning, Corning, NY, USA). The samples were surface-disinfected using a series of washes that consisted of 3% bleach (1×), sterile phosphate-buffered saline (PBS, 3×), 70% ethanol (ETOH, 1×), and finally sterile PBS (3×) to ensure removal of all disinfectants. The tick was then homogenized with a Dounce homogenizer on ice in 1 ml of media (Dulbecco's modified Eagle medium [DMEM] with 5% fetal bovine serum [FBS] and 1% HEPES) and aliquoted (250 µl) onto confluent Vero cell monolayers in a 24-well plate. After inoculation, the plates were centrifuged for 1 h at 700×*g* at 22 °C to facilitate the cell entry process. After centrifugation, the monolayers were rinsed with 1 ml of DMEM to remove any tick exoskeleton. Then 1 ml of media alone or media plus penicillin–streptomycin (50 U/ml–50 µ/ml) was added to the inoculated cell monolayers and the plates were incubated at 34 °C with 5% CO_2_. After 48 h, the medium was changed to antibiotic-free medium containing 3% FBS. The cells were monitored daily for cytopathic effect (CPE) using an inverted microscope, and the infection was confirmed using Diff-Quik staining. Wells were harvested at 70% infection and inoculated into Vero confluent 25 cm^2^ flasks and a replicate well stored at −80 °C. Cells were monitored until at least 70% infection as determined by Diff-Quik staining and spreading into Vero cells confluent in 150 cm^2^. Once the larger flask was at least 70% infected, the bacteria were further propagated, and DNA was extracted for sequencing as previously described.

## Results

**Fig. 2:**
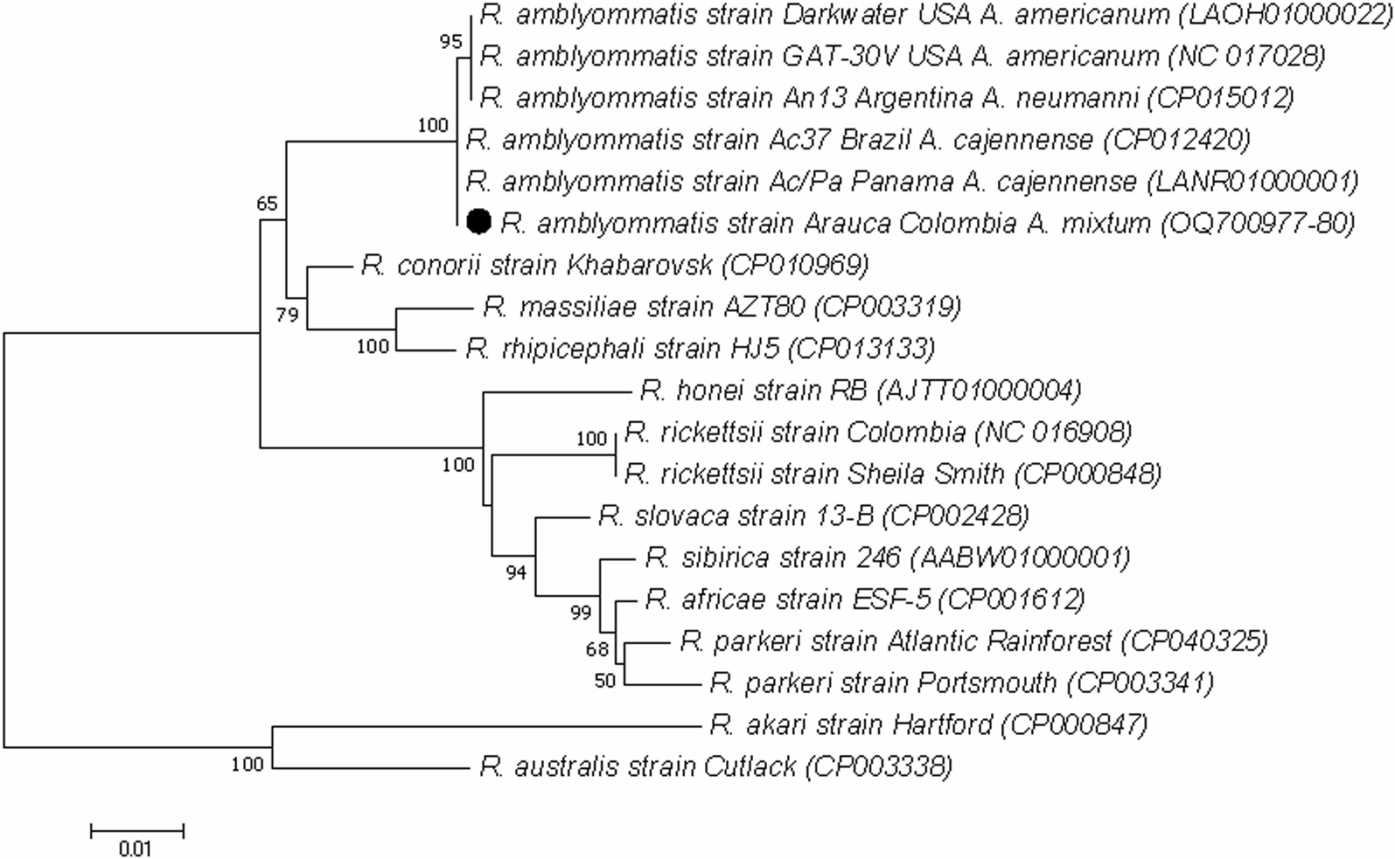
Molecular phylogenetic analysis of *Rickettsia amblyommatis* isolated from Colombia. Consensus phylogenetic tree of the concatenated *gltA*, *ompB*, *htrA*, and *ompA* genes for the sequences obtained from the *Amblyomma mixtum* isolate. The analysis was performed using the maximum composite likelihood approach with the generalized time reversible substitution model and gamma distribution. Branch supports were generated by bootstrap (1000 replicates). The tree was rooted with *R. akari* and *R. australis* species from the transitional group. A total of 19 *Rickettsia* strains were used. The final alignment presented 2104 nucleotides.

Sixty-one ticks were collected in all locations. Specimen information is presented in Table 2. The 16S mt rRNA was amplified in all samples, indicating molecular inhibitors were not present. Only one female of *A. mixtum* from Bocas del Arauca village (Arauca department and municipality, coordinates 7.015646°–70.584402°), collected on vegetation, was amplified by qPCR (*gltA*), indicating a frequency of 1.6% (1/61) for *Rickettsia* spp. infection. The isolation protocol was applied to this tick, and after three established passages, conventional PCR was performed. Sequence analysis in BLASTn showed 100% identity with *gltA* (340 base pairs [bp]), 99.87% for *ompB* (782 bp), 98.99% for *htrA* (497 bp), and 100% for *ompA* (488 bp) to different strains of *R. amblyommatis* (MH521292, KY628368, CP012420, MN336348). Concatenated phylogenetic analysis confirmed these findings, indicating that the isolate is grouped with other sequences of *Amblyomma cajennense* complex from Panama and Brazil within the *R. amblyommatis* clade (Fig. 2). The sequences obtained were deposited in GenBank with accession numbers OQ700977, OQ700978, OQ700979, and OQ700980, and the isolate was designated as *R. amblyommatis* strain Arauca and stored at the Galveston National Laboratory, TX, USA.

**Table 2:** General data of the tick species according to origin place, source, and sex

Departments	Tick species	Source	Females (no.)	Males (no.)
Arauca	*Amblyomma mixtum*	Vegetation, *Bos taurus*	7	1
Cundinamarca	*Amblyomma maculatum*	*Canis familiaris*	2	0
	*Amblyomma ovale*	*C. familiaris*	1	13
	*Amblyomma patinoi*	*C. familiaris*, *Equus asinus* × *Equus caballus*, *E. caballus*	4	5
	*Rhipicephalus sanguineus* s.l.	*C. familiaris*	11	11
Santander	*Amblyomma mixtum*	*Bos taurus*	1	0
		*E. caballus*	4	1
Total			30	31

## Discussion

According to Kaparthy et al., *R. amblyommatis* is considered the species of the SFG with the greatest distribution and prevalence in America [31]; therefore, its detection in Colombia is expected, and the present work amplifies its reports for this territory. In Colombia, there have been reports of this bacterium in *A. cajennense* s.l. and *Rhipicephalus microplus* in the department of Cundinamarca; in *Amblyomma longirostre*, *Amblyomma varium*, and *Ixodes* spp. infesting birds in the department of Caldas; and in *Amblyomma patinoi* in humans from Antioquia [32–34].

There are multiple species of hard ticks in which the bacterium has been detected. For example, in Central America, approximately 10 species are involved, with minimum infection rates up to 91% in *A. mixtum* from Panama [35, 36]. Although four species of *Amblyomma* were identified from different regions of Colombia, the percentage of natural infection of *R. amblyommatis* was only 1.6% in the ticks studied. The absence of rickettsial infection in *A. maculatum*, *A. ovale*, and *A. patinoi* could be due to the differences in the ecological conditions of each region studied for the maintenance of bacterial cycles, which in turn could have a negative impact on the ecology of rickettsiosis considering the phenomenon of transovarial interference that can occur in ticks [37]. On the other hand, the variation in hosts from which the ticks were collected could have an influence on the presence of *Rickettsia* in *Amblyomma* species, since the immunological response of each host could affect rickettsial amplification and subsequent tick acquisition [38].

In addition to molecular detection, in Colombia there is evidence of *R. amblyommatis* exposure in humans and animals, evidenced by the detection of antibodies against this species or against antigenically close species using indirect immunofluorescence assay (IFA) in human and animal samples from the departments of Antioquia and Arauca [39, 40]. This exposure is important mainly because of the role that these bacteria seem to play in rickettsiosis cycles. *Rickettsia amblyommatis* has an undetermined pathogenic role, although some studies have suggested a role in human infections. Due to antigenic cross-reaction between species of the SFG, exposure to a non-pathogenic or low to moderately virulent species can generate antibodies that can prevent serious infections by highly virulent species [37]. For this reason, it could serve as a preventive model for cases of rickettsiosis caused by *R. rickettsii*, which has also been historically reported in different Colombian regions [31, 41]. It is worth highlighting previous experimental studies with animal models that have supported this protective characteristic of *R. amblyommatis* [15, 17].

Some animal models have suggested that *R. amblyommatis* can cause disease, which has led to the hypothesis that perhaps some strains may play a pathogenic role, thus highlighting the importance of bacterial isolation and further experimental trials as well [15, 17]. The isolation of *Rickettsia* from Colombia has been limited to species such as *R. rickettsii*, *R. parkeri* strain Atlantic rainforest, and ‘*Candidatus* Rickettsia colombianensi,’ especially due to the complexity of technical and biosafety requirements [42–45]. Therefore, the first successful isolation of *R. amblyommatis* from Colombia achieved in this study contributes to the knowledge of this bacterium. This achievement will lead to the improvement and design of basic diagnostic tools, including the comparison between antigens by IFA, development of specific molecular tests, and the design of genetic, genomic, and proteomic characterization and comparison studies, among others.

### Supplementary Information

**Additional file 1: Table S1.** Location data of tick collections between 2019 and 2020 in Colombia

## Data Availability

Sequences are available in GenBank with accession numbers OQ700977, OQ700978, OQ700979, and OQ700980.
